# Value of the triglyceride–glucose index and related parameters in heart failure patients

**DOI:** 10.3389/fcvm.2024.1397907

**Published:** 2024-07-18

**Authors:** Yunteng Fang, Jiayi Shen, Lingchun Lyu

**Affiliations:** ^1^ Lishui Hospital, Zhejiang University School of Medicine, Hangzhou, China; ^2^ Lishui Central Hospital and the Fifth Affiliated Hospital, Wenzhou Medical University, Lishui, China

**Keywords:** TyG index, heart failure, obesity paradox, SGLT-2 inhibitors, HOMA-IR

## Abstract

The triglyceride–glucose (TyG) index, proven to be a crucial insulin resistance biomarker (better than the Homeostasis Model Assessment for Insulin Resistance), is simple and non-invasive. Recently, indisputable evidence has shown that the TyG index is strongly associated with cardiovascular disease [CVD, including atherosclerosis, heart failure (HF), and hypertension] prognosis and mortality. Nevertheless, the value of the TyG index in HF patients treated with sodium–glucose cotransporter 2 inhibitors (SGLT2is) has not been systematically evaluated. Therefore, in this review, we summarized the value of the TyG index and its related parameters as markers of CVD, especially HF. Furthermore, we addressed the use of SGLT2is and GLP-1 receptor antagonists in HF patients. Finally, we summarized the mechanism of the “obesity paradox.”

## 1 Introduction

Heart failure (HF), including HF-related symptoms and signs, is a complex clinical syndrome that results from certain functional or structural damage to the ventricles, impairing their ability to fill with or eject blood (1). Approximately 40 million people worldwide suffer from HF, which can be caused by many factors, such as hypertension, coronary artery disease (CAD), smoking, obesity, and genetic factors (2). HF is a leading cause of hospitalizations in patients aged >65 years, and 1%–2% of all hospitalizations in the Western world are attributed to HF (3). In addition, a study conducted in China indicated that HF poses a substantial burden on health systems, and people may face more serious challenges in the future. In the coming years, the epidemic of HF is possibly to worsen, mostly on account of the millions of patients with increasing risk factors, such as coronary heart disease, hypertension, and diabetes. In addition, the figures of this study show that the prevalence and incidence of HF increase with age. With the trend of an aging population, the total number of HF patients is growing rapidly, especially in China, which has the largest population worldwide (4). HF can result in many wasted resources and other types of losses—premature death, lost productivity, disability, loss of quality of life, and racial health disparities (5); thus, there is an urgent need to address this problem. Therefore, to better stratify and cope with the devastating risks of HF, a multitude of biomarkers that can be obtained with non-invasive methods have been identified. For instance, B-type natriuretic peptide (BNP) and N-terminal proBNP (NT-proBNP) have worldwide applicability and high diagnostic and therapeutic validity for the treatment of HF (6). Molvin et al. reported that mid-regional pro-adrenomedullin (MR-proADM) and NT-proBNP concentrations are positively correlated with the risk of experiencing postdischarge mortality, with NT-proBNP being the exclusive biomarker for predicting cardiac hospitalizations (7). It remains unclear which is the best indicator for HF or which combination of several indices is better. NT-proBNP is stable in circulation, while BNP is affected by renal function and age. Interestingly, in clinical practice, BNP is prone to more frequent testing due to its shorter half-life, and it is a widely recognized biomarker.

**Figure 1:**
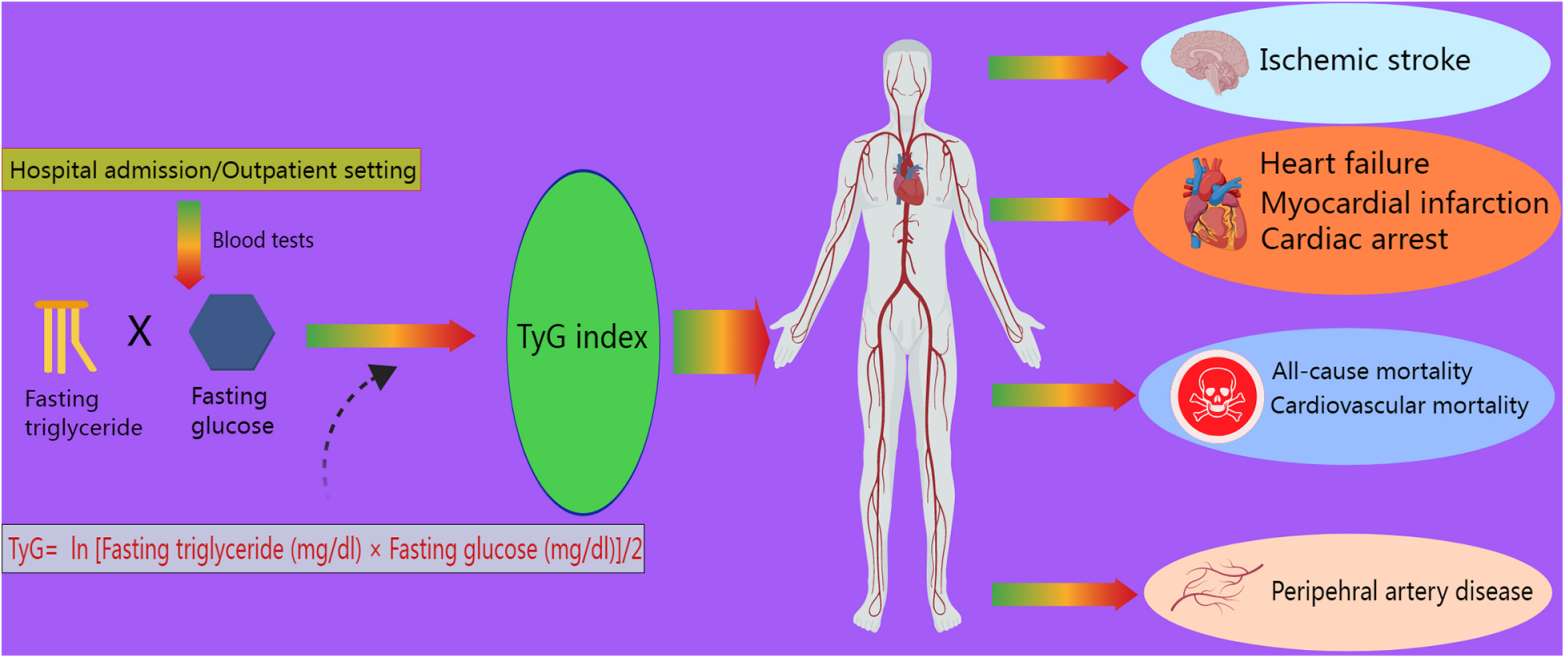
Role of the TyG index in cardiovascular outcomes. The TyG index, defined as ln [fasting triglyceride (mg/dl) × fasting glucose (mg/dl)]/2, is measured during admission or outpatient visits. It is associated with ischemic stroke, myocardial infarction, heart failure, cardiac arrest, peripheral artery disease, cardiovascular mortality, and all-cause mortality.

However, with ongoing research, more novel biomarkers have been utilized as predictors in clinical practice. The triglyceride–glucose (TyG) index, an indicator of insulin resistance (IR), calculated as TyG index = ln [fasting triglyceride (mg/dl) × fasting glucose (mg/dl)]/2, was independently associated with a higher incidence of acute kidney injury (8), CAD, and atherosclerotic cardiovascular diseases, such as myocardial infarction (MI) and stroke, in a meta-analysis involving 5,731,294 participants (9). Moreover, the TyG index has been shown to correlate with several cardiovascular outcomes, such as cardiac arrest and peripheral artery disease (10, 11) (Figure 1). Hence, according to recent studies, this index is strongly associated with the risk of developing cardiovascular disease (CVD), especially HF. As a promising prognostic marker of HF with preserved ejection fraction (HFpEF), a high TyG index is closely associated with an increased risk of mortality and rehospitalization in patients with HFpEF (12). In addition, the TyG index, a valuable tool for use in individuals with prediabetes or diabetes, was associated with the risk of experiencing all-cause and CVD mortality (13) and the risk of experiencing subclinical HFpEF in patients with type 2 diabetes (T2DM) (14). The underlying mechanism is that microRNA 181c, which targets cardiac fibroblasts, is elevated in frail elderly adults with diabetes (15). For patients with HFpEF, insulin resistance and the TyG index are important, as a study revealed that insulin resistance is associated with myocardial dysfunction in HFpEF patients (16). Concurrently, the term “metabolic cardiomyopathy,” referring to the condition under which metabolic inflammatory molecules damage myocardial function in patients with insulin resistance and diabetes, has been gradually used in the literature on HFpEF (17). In most studies, the TyG index is usually measured at admission to better predict the incidence or prognosis of HF. However, a study also collected data in the outpatient setting (18). It was also reported that the TyG-BMI (body mass index), a parameter related to the TyG index and connected with IR, is significantly associated with the risk of developing hypertension (19). The above-mentioned studies show that the TyG index is closely associated with CVD incidence and mortality; thus, it is reasonable to focus on the relationships between HF incidence and the TyG index and its related parameters. Recently, as part of the effort to cure HF, several new drugs have been developed. Sodium–glucose cotransporter 2 inhibitors (SGLT2is) and GLP-1 receptor agonists (GLP-1 RAs) are known to diminish cardiovascular risks in patients with T2DM (20). These popular drugs were initially targeted at treating diabetes, but various studies have shown that they have unexpected effects on HF and have gradually been confirmed to reduce HF-related mortality.

Hence, in this review, we aimed to discuss the usefulness of the TyG index compared to that of the Homeostasis Model Assessment for Insulin Resistance (HOMA-IR) for identifying IR. In addition, we reviewed the published literature that highlights the application value of NT-proBNP, MR-proADM, the TyG index, and related parameters in HF. Furthermore, we explored the effects of the increasingly popular SGLT2is on HF and their correlation with the TyG index.

### 1.1 Comparison of the TyG index with the HOMA-IR

HOMA-IR, determined as [fasting insulin (μIU/ml) × fasting glucose (mmol/L)]/22.5, is a marker that reflects IR and can be simultaneously quantified by the TyG index in some metabolic diseases that increase the risk of cardiovascular disease (21). When comparing the TyG index with the HOMA-IR, the former was found to be more valuable for identifying IR in Chinese T2DM patients with a BMI less than 35 kg/m^2^ (22). A study enrolling 3,185 patients with T2DM showed that each one-unit increase in the TyG index was accompanied by a 1.40-fold increase in the incidence of progressive arterial stiffness. Nonetheless, there was no notable association between HOMA-IR and the incidence of progressive arterial stiffness (23). Moreover, Park et al. analyzed data from 9,730 adults and reported that the TyG index was better able to predict the incidence of T2DM than HOMA-IR, with an area under the curve (AUC) of 0.640 (0.628–0.652) for the incidence of T2DM predicted by the TyG index and 0.531 (0.521–0.541) for the incidence of T2DM predicted by HOMA-IR (24). Moreover, Son et al. (25) conducted a study of 6,091 adults without metabolic syndrome (MetS) and demonstrated that HOMA-IR had a less powerful ability to predict the prevalence of MetS than the TyG index. The results of the Coronary Artery Risk Development in Young Adults investigation, which involved 4,992 participants, suggested that the TyG index can function as a surrogate biomarker for IR to predict the risk of chronic HF (CHF), potentially replacing HOMA-IR (26). As Wang et al. stated, the TyG index presented a stronger connection with intracranial atherosclerosis than HOMA-IR. The authors reported that the TyG index reflects insulin resistance not only in hepatic tissues but also in peripheral tissues, while the HOMA-IR index reflects insulin resistance in only hepatic tissues (27). In addition, the TyG index accounts for both lipids and glucose, making it more appropriate than HOMA-IR for predicting atherosclerosis. Beyond atherosclerosis, an inverse association was observed between HOMA-IR and cognitive function in older patients with hypertension and prediabetes (28). Previous studies have proven an association between renal damage and cognitive function in frail, hypertensive older adults with diabetes (29). In this way, insulin resistance may correlate with cognitive impairment in populations with diabetes and have potential effects on renal function. Similarly, the TyG index has been proven to correlate with low cognitive function in non-diabetic elderly patients (30). The strength of these studies lies in the elucidation of the relationship between cardiovascular damage and cognitive impairment. However, no study has compared the two metabolic parameters in the investigation of cognitive function. In short, the TyG index is more useful than HOMA-IR for the diagnosis of CVD and MetS. Notably, many authors have ascertained the usefulness of the TyG index in numerous studies, but few have noted its ability to predict disease. The TyG index is a relatively comprehensive index since it is a composite of lipids and glucose, but HOMA-IR still has a unique value. In future studies, more diseases, including dementia, cancer, and mental disease, could be used for further comparison of these two parameters.

The TyG index is not superior to HOMA-IR in every aspect. For example, Liu et al. showed that the ability of the TyG index to predict testosterone deficiency was actually inferior to that of HOMA-IR (31). In this way, HOMA-IR may have unexpected impacts on conditions other than cardiovascular disease, warranting further studies. However, overall, the TyG index is more effective than the HOMA-IR index in diverse aspects if applied pertinently. Further exploration into the strengths or shortcomings of the TyG index is expected in future research.

### 1.2 The TyG index is linked to mortality

Several studies (13, 32, 33) concluded that the TyG index was independently associated with both all-cause mortality and cardiovascular mortality. Cai et al. (34) reported that an elevated TyG index was significantly associated with hospital mortality and ICU mortality. Similarly, a study involving 3,026 patients (35) revealed that the TyG index independently predicted the risk of hospital death and ICU mortality, with each one-unit increase in the TyG index accompanied by a 1.19-fold increase in the risk of death during hospitalization. Zhang et al. (36) reported that the TyG index was independently associated with an increased risk of ICU mortality and hospital mortality. Indeed, in view of the general population, a positive relationship was clearly found between the TyG index and cardiovascular mortality in people under 65 years old (37). That meta-analysis presented a shortcoming in that the TyG index measurement methods and study designs were different, which might have led to heterogeneity. Obviously, the TyG index plays a potential role in predicting the mortality rate according to all convincing studies that have carefully considered different mortality types. Apart from the above mortality-related findings, Yang et al. emphasized in a meta-analysis (38) that among ischemic stroke patients, a higher TyG index was associated with a higher risk of experiencing stroke recurrence or mortality. This study revealed that the TyG index might have tremendous predictive ability for various vascular diseases throughout the body, a condition referred to as “panvascular disease.” Glucose in the blood can trigger inflammation in vessels, leading to stenosis and stiffness and increasing the susceptibility of patients with diabetes to CVD and cardiovascular mortality. Panvascular disease refers to atherosclerosis that usually occurs in large vessels and microvessels, including renal, cerebral, peripheral, and cardiac vessels, in patients with diabetes (39). Hence, when we determine the function of the TyG index in CVD mortality, it is suitable to apply it to other vascular diseases, especially under conditions of impaired glucose tolerance. However, the available literature mostly focuses on the association between CVD mortality and a single vascular lesion and barely concerns the multifactorial atherosclerosis burden; thus, researchers should further explore these associations in patients with panvascular disease. Moreover, most current studies are retrospective and cannot be used to determine causality in the association between the TyG index and adverse CVD outcomes. The URRAH cohort, a prospective study including 16,649 participants, indicated that a higher risk of all-cause and CV mortality was associated with an increased TyG index (40). The strengths of this research were the long follow-up of 144 months and the large and homogeneous general population. In a state of insulin resistance, the activation of oxidative stress and the inflammatory response may damage cardiomyocytes, and hyperglycemia and inflammation can subsequently lead to and predict the occurrence of adverse cardiovascular events (41). Hence, this prospective study strongly demonstrated that a higher TyG index reflecting anomalies of lipid and glucose metabolism is a risk factor for predicting adverse CVD outcomes in a causal relationship.

Despite the positive association between the TyG index and mortality reported in numerous remarkable studies, the TyG index seemingly does not correlate with cardiovascular mortality (3 studies) or all-cause mortality (4 studies), according to a meta-analysis of a collection of 12 cohort studies (42). Moreover, a contradictory study (32) argued that at the 1-year follow-up, there was no difference in the TyG index among patients with or without major adverse cardiovascular events (MACEs) [8.73 (8.36–9.08) vs. 8.81 (8.5–9.17); *P* = 0.09]. Therefore, the authors concluded that the use of the TyG index to predict MACEs and overall mortality in non-diabetic individuals with MI was not advisable. The reasons for these inconsistent results lie in the limited sample sizes and differences in ethnicity since meta-analyses combine many cohort studies and might amplify the likelihood of statistical significance, whereas a single study may fail to achieve these results. In addition, many studies did not provide sufficient follow-up time, and the TyG index was measured only once, which might also slightly affect the statistical power.

### 1.3 The value of NT-proBNP and MR-proADM in HF

NT-proBNP plays a key role in the development of HF. In patients with HF with mildly reduced ejection fraction (HFmrEF) or HFpEF, a much greater risk of experiencing cardiovascular events was consistently associated with increased NT-proBNP (43). With respect to the risk of obesity, increasing NT-proBNP was independently associated with a greater risk of cardiac death in all BMI groups except for people who were severely obese (BMI ≥ 40 kg/m^2^) (22). The authors of that study wisely adjusted for obesity status, which is a crucial cause of CVD-related death, and promoted the reliable prognostic value of NT-proBNP. In a US population trial, Ciardullo et al. (44) reported that participants with NT-proBNP levels ≥100 pg/ml suffered a greater incidence of both cardiovascular mortality and all-cause mortality. These results verified that NT-proBNP is indispensable for predicting incidence and mortality in HF patients. Of course, in clinical practice, NT-proBNP levels less than 100 pg/ml cannot be arbitrarily used to exclude HF without considering symptoms or other tests.

In addition to mortality, NT-proBNP is also beneficial for assessing treatment effects. Based on what Ezekowitz et al. explained, compared to that of placebo, the impact of vericiguat on the endpoint was more pronounced in patients with NT-proBNP levels less than 8,000 pg/ml, and this effect was further amplified in patients with NT-proBNP levels less than 4,000 pg/ml (45). In turn, Armstrong et al. reported that compared to those taking a placebo, patients treated with vericiguat experienced more significant decreases and fewer increases in sequential measures of NT-proBNP, and these interesting alterations were seemingly related to the advantages of vericiguat therapy (46). Therefore, the causality of the relationship between vericiguat treatment and the level of NT-proBNP remains unclear because of the limitations of retrospective studies. Medicinally controlled or lower NT-proBNP is sensitive to treatment. Notably, in an important study, Cunningham and Myhre (47) argued that NT-proBNP is an imperfect surrogate to assess the response to HF therapies because a convincing sensitivity analysis, which exclusively included patients with an ejection fraction of less than 30%, revealed no relationship between treatment effects and NT-proBNP concentrations.

In a different study, researchers aimed to evaluate the effects of combinations of indices. The combined use of MR-proADM and NT-proBNP measurements had additional predictive value mortality and new readmission for acute HF after discharge at 30 days (48). Surprisingly, based on a randomized controlled trial (49) involving 214 patients, Klip et al. reported that MR-proADM (AUC = 0.81) demonstrated greater predictive strength for mortality than BNP (AUC = 0.66) and NT-proBNP (AUC = 0.67). Furthermore, compared with both BNP and NT-proBNP concentrations, MR-proADM concentrations markedly improved mortality risk prediction. This finding suggested that NT-proBNP is not the best predictor of HF since there are other biomarkers that provide better precision and accuracy. Fraty et al. concluded that MR-proADM serves as a prognostic biomarker for HF in people with T2DM; however, compared with NT-proBNP, it does not provide significant complementary information on the prediction of HF without T2DM (50). An apparent shortcoming of this study was its failure to establish a solid history of CHF and a lack of cardiac ultrasound information, which included important baseline left ventricular ejection fractions. The authors thought that diabetes status would affect outcomes in some situations, but echocardiography was not easily available for patients without diabetes. Hence, this study lacked abundant information on non-diabetic control groups.

Five different types of failure—respiratory system, coagulation system, cardiovascular system, neurological system, and kidney organ failure—were independently predicted by MR-proADM (51). In clinical practice, MR-proADM is not widely used by doctors as a standard predictor of HF since this index is still immature or unreliable, but its value can be exploited. A study enrolling 1,088 patients with CHF (52) indicated that patients with elevated MR-proADM levels tended to be older, have a higher New York Heart Association (NYHA) class, be frequently afflicted with lower limb edema, and have a greater incidence of chronic diseases such as hypertension, diabetes, and atrial fibrillation. However, MR-proADM has a poor ability to confirm or exclude HF (53). MR-proADM is not commonly used to evaluate HF, so further prospective studies are warranted. Clearly, with the growing need to reduce the mortality of HF patients, more prognostic and predictive biomarkers need to be explored and updated.

### 1.4 The value of the TyG index in HF

In HF patients, the TyG index is reportedly related to mortality Table 1 (1, 12, 33, 35, 54–57). Among patients with HFpEF, a high TyG index, as a study enrolling 823 patients claimed, is associated with an elevated risk of mortality and rehospitalization (12, 14). The TyG index not only predicts HFpEF but also acute decompensated HF (ADHF) due to the evidence from a paper elaborating that an elevated TyG index is independently linked to an unfavorable prognosis, making it a valuable factor for stratifying the risk of patients with ADHF (37). Similar results were reported by Guo et al. regarding CHF (58). Zheng et al. also reported in a prospective study that an elevated risk of HF is associated with a high cumulative TyG index (59). In China, a study reported that in groups in which the highest and lowest TyG index tertiles were compared, the hazard ratios (HRs) for all-cause and CV-related deaths were 1.84 and 1.94, respectively (33). In addition, a significant positive association existed between HF incidence and the TyG index, which concurrently was proven to be connected with an elevated incidence of coronary heart disease, dyslipidemia, and hypertension (60).

**Table 1:** Association between the TyG index and mortality of patients with HF.

Author	Year	Study design	Study size	Key findings
Zhou et al. (12)	2023	Prospective study	823	TyG index was associated with the incidence of all-cause death (HR: 1.46, 95% CI: 1.10–1.96, *P* = 0.009) and CV mortality (HR: 1.49, 95% CI: 1.13–1.97, *P* = 0.005)
Zhou et al. (33)	2023	Retrospective study	6,697	TyG index was associated with all-cause death (HR: 1.84, 95% CI: 1.61–2.10; *P* for trend < 0.001) and CV death (HR: 1.94, 95% CI: 1.63–2.30; *P* for trend < 0.001)
Liao et al. (35)	2022	Retrospective study	3,026	TyG index was a risk predictor of ICU death (HR: 1.72, 95% CI: 1.18–2.52, *P* = 0.005) and hospital death (HR: 2.19, 95% CI: 1.59–3.03, *P* < 0.001)
Han et al. (54)	2022	Retrospective study	4,411	TyG index was associated with in-hospital mortality (OR: 1.886, 95% CI: 1.421–2.501, *P* < 0.001)
Cheng et al. (55)	2024	Retrospective study	886	TyG index levels raised the risk of death in the hospital (OR: 1.73, 95% CI: 1.03–3.27, *P* = 0.031)
Özcan et al. (56)	2023	Retrospective study	773	The mortality rates were 14.0% (HR: 2.24, 95% CI: 1.42–6.88) in TyG index tertile 2 and 23.3% (HR: 3.88, 95% CI: 1.84–14.38) in TyG index tertile 3
Sun et al. (57)	2023	Retrospective study	2,055	TyG index was independently associated with all-cause mortality (HR: 2.18, 95% CI: 1.50–3.18; *P* < 0.001, *P* for trend < 0.001)

CI, confidence interval.

According to the findings from Sun et al., an independent and positive correlation between the TyG index and the risk of experiencing MACEs was detected among patients with HF undergoing percutaneous coronary intervention (57). Hao et al. concluded that the TyG index is closely associated with the risk of experiencing HF and is likely a valuable indicator for predicting long-term patient prognosis in individuals with acute MI (AMI) (61). Notably, Huang et al. reported that a 0.1-unit increase in the TyG index was associated with a 1.05-fold increase in the risk of experiencing MACEs and a 1.07-fold increase in the risk of worsening HF (62). Confirmed by evidence from two large cohorts (63), Li et al. explained that, a higher TyG index, serving as an independent as well as causal risk factor, contributed to incident HF in the general population. This study included two large-sample-size general population cohorts and utilized Mendelian analysis. In addition, this study clarified the causal relationship between the TyG index and the incidence of HF. However, it is important to note that the Mendelian analysis was limited by its exclusive use of data from patients of European descent, which makes it difficult to extrapolate the results to other populations.

No matter how valuable this index is, there are always limitations and failures. For example, Li et al. reported no meaningful association between the TyG index and the risk of experiencing HF in individuals with a BMI of less than 30 kg/m^2^ (60). In fact, the outcome was triggered by numerous factors. First, the study excluded patients whose HF was not officially diagnosed, and the selection population may have bias. Second, some patients without the TyG index and HF data were excluded. Third, because of the lack of generalizability, this study failed to maintain accuracy in other regions. In the same study, Li et al. concluded that an elevated risk of stroke was not significantly associated with the TyG index, while Zhao et al. (64) showed that the TyG index can independently predict the onset of ischemic stroke in the general population. This discrepancy resulted from the fact that Zhao et al. enrolled only people older than 40 years in rural China in their prospective cohort study, so the range and ethnicity were disparate.

### 1.5 The TyG index and SGLT2is in HF

Not until guideline-directed medical therapy (GDMT) for HF with reduced ejection fraction (HFrEF) recommended four medication classes, including SGLT2is, did people widely apply SGLT2is in clinical practice (1). Through many retrospective and prospective studies, SGLT2is have been recognized as safe and absolutely effective in the treatment of HF in many situations and have emerged as a miraculous crossover drug. According to a meta-analysis of 8,474 patients (65), SGLT2is contributed to a greater than 25% reduction in the combined risk of cardiovascular death or initial hospitalization for HF. In addition, they decreased the risk by 25% in the composite of recurrent hospitalizations for HF. In addition, it was reported that among patients with HF, HFpEF, and HFmrEF, dapagliflozin decreased the combined risk of worsening HF and cardiovascular death (66). Three studies (67–69) focused on one famous trial, the EMPEROR-Preserved Trial involving 5,988 patients, which suggested that empagliflozin reduced the risk and severity of a large range of worsening HF events, whether in or out of the hospital, and decreased the risk of HF consequences irrespective of diabetes status. Consistently, Butler et al. asserted that compared with placebo treatment, empagliflozin treatment resulted in a comparable reduction in the risk of cardiovascular death or hospitalization for HF in both sexes (70). Overall, the above-mentioned results elucidated the meaningful use of SGLT2is in HF and can guide clinical therapy to effectively minimize HF risk and mortality. Paradoxically, a multicenter, double-blinded trial (71) showed that dapagliflozin and ertugliflozin were completely non-inferior to placebo for reducing the risk of experiencing MACEs. However, the authors failed to explain the outcomes robustly and suspected that the trial may have been influenced to a greater extent by the evolving long-term changes in more intensive secondary preventive therapies in recent years compared to earlier trials.

Based on certain studies, the TyG index is used as an indicator to assess the effects of SGLT2is in some respects. Zhu et al. (72) reported that dapagliflozin was strongly associated with a decreased risk of experiencing MACEs and that the TyG index decreased with dapagliflozin administration. This study suggested that dapagliflozin affects both lipid metabolism and glucose metabolism, which is comprehensively reflected by the TyG index. This finding provided enough evidence that the TyG index can be utilized as an easily accessible indicator in clinical practice to evaluate the curative effects of dapagliflozin, and it is not just an index to predict mortality. Nonetheless, missing measured factors at discharge and longer follow-up were shortcomings of that study. As indicated by various plasma atherogenic biomarkers, both empagliflozin and dapagliflozin treatment have been demonstrated to result in substantial alterations in the TyG index regardless of the administration of statin treatment (73, 74). From this perspective, the usefulness of the TyG index extends beyond the indication of HF, as it can also be used to evaluate some medical effects, which is another pleasant surprise for physicians. In conclusion, after receiving more research attention, the TyG index will be fully applied in clinical work. There are a dozen indices that can be used to assess therapeutic effects, but the TyG index is simple and quick.

### 1.6 GLP-1 RAs and HF

GLP-1 RAs are new drugs that are characterized by amazing glucose-lowering effects. Initially, people often applied them for diabetes treatment and blood glucose control; however, spectacular unexpected effects on the heart have been investigated recently. In a meta-analysis (75) involving 60,080 patients, GLP-1 RAs reduced the risk of experiencing all-cause mortality by 12% and hospital admission for HF by 11%, indicating that GLP-1 RAs are cardioprotective, similar to SGLT2is. Moreover, Zhao et al. reported that compared with placebo, GLP-1 RAs may significantly lower the risk of experiencing MACEs in individuals with HF (76). Apart from the respective contributions of SGLT2is and GLP-1 RAs, researchers have tested whether their combination yields better results. Compared with other combination regimens, a 57% lower odds of HF was significantly associated with the combination of SGLT2is and GLP-1RA regimens (77). However, the mechanism of action of GLP-1 RAs is not completely clear. Recently, Ussher et al. reported that by targeting the central nervous system, people taking GLP-1 RAs tend to have a decreased appetite, leading to weight loss and improved cardiovascular outcomes. In addition, to reduce hepatic steatosis, GLP-1 RAs indirectly improve circulating lipid profiles. GLP-1 RAs might also affect blood vessels, kidneys, and the heart to prevent atherosclerosis and ameliorate cardiac function (78). In addition, a lower risk for hospitalization for HF was associated with SGLT-2 inhibitors than with GLP-1 RAs (79). Interestingly, that meta-analysis included 23 cardiovascular outcome trials, with a considerable sample size and minimal risk of bias. However, no trial has directly compared antidiabetic drug classes, so the meta-analysis failed to interpret the data based on direct comparisons. The above studies highlighted that GLP-1 RAs are extraordinary novel drugs for the treatment of HF, albeit inferior to SGLT2is.

However, Merza et al. noted that compared with a placebo, the controversial medicine GLP-1 RAs did not increase the risk of experiencing MACEs (80). However, in his study, Merza et al. assembled only 9 randomized controlled trials (RCTs) containing only 871 participants, which is small for a meta-analysis. Moreover, RCTs are too limited to produce accurate outcomes in contrast with real-world data; thus, more prospective studies are needed in authentic situations in the future.

### 1.7 Related parameters of the TyG index and the obesity paradox in HF

Some derivative indices, such as the waist-to-height ratio (TyG-WHtR), the waist-to-height ratio (TyG-BMI), and the waist circumference (TyG-WC), have also attracted attention for their ability to predict CVD (81) in certain ways since they are more comprehensive. These related indices contribute to the prognosis of HF or any other CVD, such as the TyG index. Among them, the TyG-BMI is widely studied because weight and height are essential for assessing the risk of CVD. For example, a lower TyG-BMI was linked to a lower incidence of HF in both prediabetes mellitus and diabetes mellitus patients (82). In addition to the TyG-BMI, Dang et al. reported that the TyG-WC and TyG-WHtR are correlated with congestive HF, which proved the significance of these two indices (83). From this perspective, the TyG index seems to have immense potential to indicate CVD and its combination indices also display vivid potential.

**Figure 2:**
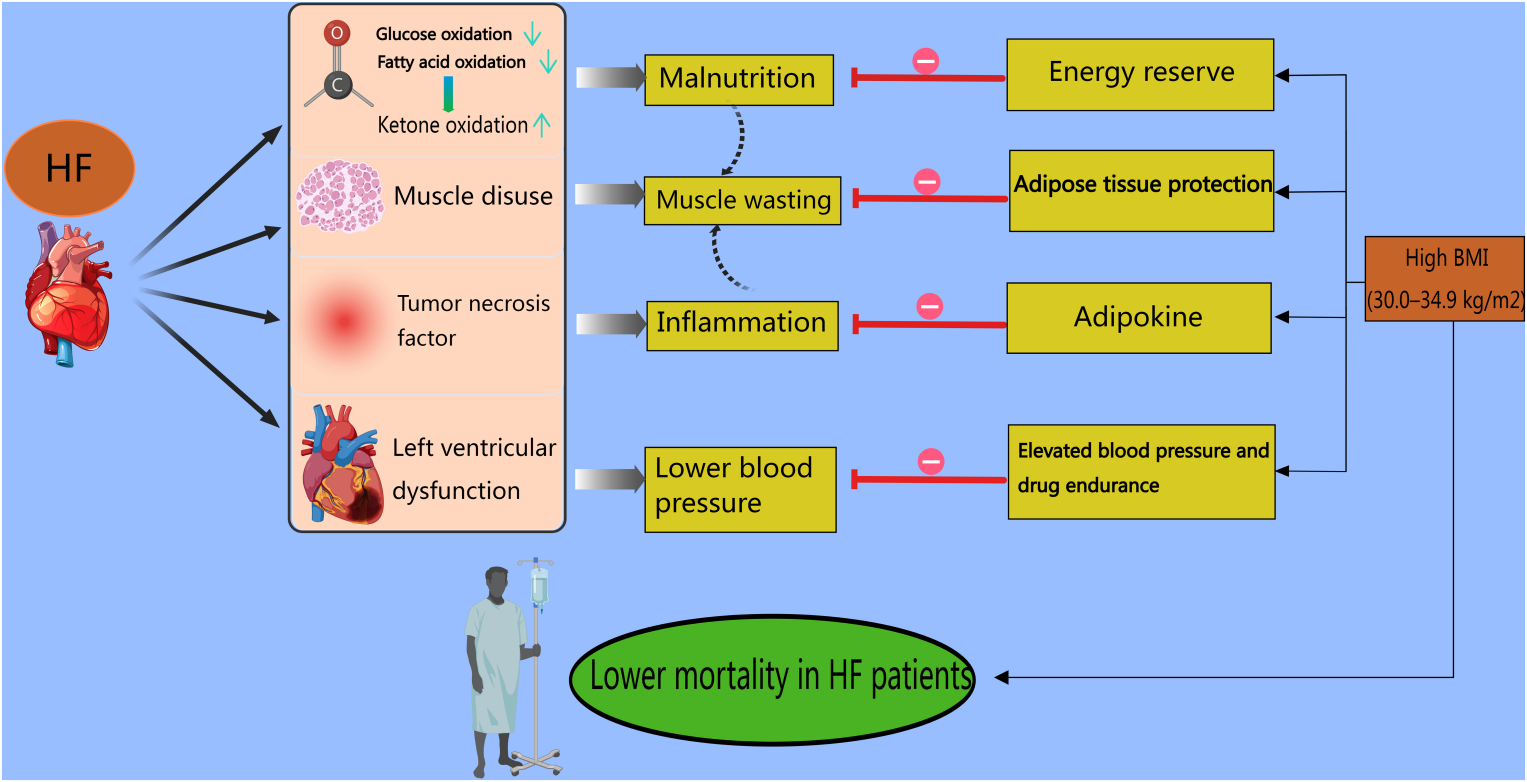
Obesity paradox in heart failure patients. In HF patients, first, fatty acid oxidation and glucose oxidation are suppressed, leading the bodies to switch to ketone fuel utilization in an energy-starved state. Second, HF patients with a deficiency of left ventricular function usually experience lower blood pressure. Third, HF patients generate tumor necrosis factor, which causes inflammation in the myocardium. Finally, muscle disuse, malnutrition, neurohumoral factor activation, and HF medications contribute to muscle wasting. The above-mentioned factors result in higher mortality rates. However, for higher-BMI (30.0–34.9 kg/m^2^) patients with HF, stored adipose tissue provides abundant energy to avoid malnutrition. In addition, obese patients present with elevated blood pressure and spare more space for drugs to target HF. Also, molecules and adipokines, provided by increased levels of cholesterol and serum lipids or hyperlipidemia, prevent the inflammatory response. Moreover, adipose tissue protects muscle. All these points counteract the damage caused by lower BMI in HF patients and result in higher mortality rates.

Regarding mortality, a study enrolling 423 patients with HF confirmed that the population with a higher TyG-BMI had a markedly greater 1-year survival rate (84). In addition, Chen et al. reported a negative correlation between the TyG-BMI and early-onset HF, whose incidence rates were explicitly lower in males than in females (14.8% vs. 29%) (85). Obviously, the finding that the TyG-BMI is positively related to the incidence but negatively related to the mortality of HF is confusing. A meta-analysis reported that HF mortality displays a U-shaped curve with a nadir at 30.0–34.9 kg/m^2^, indicating that compared with lean patients, higher-BMI patients have a survival benefit (86). Indeed, the “obesity paradox” may be a gorgeous explanation concerning the catabolic state, hemodynamic properties, adipokine protection, and muscle wasting (84). First, an “energy-starved” heart is produced by metabolic deficits in both fatty acid oxidation and glucose metabolism in HF, and this condition is primed to use alternative adenosine triphosphate (ATP)-generating fuels. Under these circumstances, individuals choose to switch to ketone fuel utilization since in HF ketone oxidation can bypass not only the complex dysregulation of the β-oxidation pathway but also the pyruvate dehydrogenase complex (87), causing malnutrition and a worse prognosis. Second, HF with a deficiency of left ventricular function usually results in lower blood pressure, and a systolic blood pressure less than 130 mmHg is associated with poor outcomes among hospitalized patients with HFrEF (88). The hemodynamics of obesity accompanied by elevated blood pressure counteract weak cardiac function and spare more space for drugs to target HF. Third, in patients with HF, cachexia can cause the generation of tumor necrosis factor (TNF), which is detrimental to the myocardium and induces inflammation. More molecules and adipokines, provided by increased levels of cholesterol and serum lipids or hyperlipidemia, bind to endotoxins and remove them from blood circulation to prevent the inflammatory response (89). Fourth, the factors involved in muscle wasting are intricate and include disuse, malnutrition, neurohumoral factor activation, and HF medications (90). In an animal trial in lean septic mice, muscle wasting and weakness were confirmed to be protected against by increased lipid availability (91). Given that adipose tissue protects muscle, underweight individuals suffer from muscle wasting, which is an independent predictor of death in patients with HF (92). Accordingly, Ohori et al. asserted that in patients with muscle wasting, the incidence of NYHA functional class III was greater (93) (Figure 2). Not surprisingly, the underlying mechanism of the “obesity paradox” is controversial and unclear because of the different outcomes reported by Benn et al. (94). Both HF incidence and mortality are causally increased by high BMI. The debates on this topic are ongoing because of many confounding factors, such as age, comorbidities, and pharmacotherapy. Multiple studies have confirmed the association between high BMI and mortality in HF patients. According to a meta-analysis involving 5,819 patients with chronic HF, the outcomes of all-cause mortality or HF hospitalization were poorer in the lower-BMI groups (95). In addition, Jones et al. emphasized that compared with people with normal weight, those with higher weight were at elevated risk of mortality (96). In the future, more accurate and comprehensive studies are required to prove this theory to better understand the mechanism of HF and develop corresponding therapies.

## 2 Conclusion

Overall, several indices can be used to predict HF, which was previously diagnosed using BNP and NT-proBNP, with positive feedback from clinicians. Gradually, physicians have attached immense importance to the TyG index, an indicator of IR, to comprehensively estimate the incidence or mortality of CVD and the progression of CAD. With the prevalent administration of SGLT2is in HF treatment, the TyG index, which includes glucose and triglycerides, shows a strong association with therapeutic effects as a non-invasive and convenient indicator. However, further studies should elucidate the underlying biological mechanism of the TyG index in different HF types. Given that the TyG index is a useful predictor and tool for stratifying the risk for stroke, fatty liver, and acute kidney injury, more studies are warranted to explore the associations of the TyG index with other targeted organs with or without HF. In addition, large cohort studies covering the general population are required to validate previously published findings. Current studies rarely consider lifestyle factors (smoking, drinking, and diet), medication treatment, and many other confounding factors, indicating the need for a full-scale study design.
